# Metabolome and Transcriptome Reveal Novel Formation Mechanism of Early Mature Trait in Kiwifruit (*Actinidia eriantha*)

**DOI:** 10.3389/fpls.2021.760496

**Published:** 2021-11-19

**Authors:** Guanglian Liao, Qing Liu, Xiaobiao Xu, Yanqun He, Yiqi Li, Hailing Wang, Bin Ye, Chunhui Huang, Min Zhong, Dongfeng Jia

**Affiliations:** ^1^Jiangxi Provincial Key Laboratory of Silviculture, College of Forestry, Jiangxi Agricultural University, Nanchang, China; ^2^College of Agronomy, Kiwifruit Institute of Jiangxi Agricultural University, Jiangxi Agricultural University, Nanchang, China

**Keywords:** *Actinidia eriantha*, early mature trait, non-target metabolomics, sucrose metabolism, transcriptomics, resequencing

## Abstract

Kiwifruit (*Actinidia eriantha*) is a peculiar berry resource in China, and the maturation period is generally late. Fortunately, we found an early mature *A. eriantha* germplasm. In order to explore the formation mechanism of its early mature trait, we determined the main carbohydrate and endogenous hormone content of the fruit, and used off-target metabolomics and transcriptomics to identify key regulatory metabolites and genes. We found that early mature germplasm had faster starch conversion rate and higher sucrose, glucose, and fructose content when harvested, while with lower auxin (IAA), abscisic acid (ABA), and zeatin (ZR) content. Through the non-targeted metabolome, 19 and 20 metabolites closely related to fruit maturity and early maturity were identified, respectively. At the same time, weighted correlation network analysis (WGCNA) showed that these metabolites were regulated by 73 and 99 genes, respectively, especially genes related to sugar metabolism were mostly. Based on above, the formation of early mature trait of *A. eriantha* was mainly due to the sucrose decomposition rate was reduced and the soluble solid content (SSC) accumulated at low levels of endogenous hormones, so as to reach the harvest standard earlier than the late mature germplasm. Finally, ten single nucleotide polymorphism (SNP) loci were developed which can be used for the identification of early mature trait of *A. eriantha*.

## HIGHLIGHTS

- The formation mechanism of early mature trait of kiwifruit (*Actinidia eriantha*) has been studied for the first time.

## Introduction

Kiwifruit (*Actinidia eriantha*) is a unique germplasm resource in China and widely distributed in the Yangtze River basin, the Yunnan–Guizhou plateau, and the Sichuan basin, at altitudes 200–2,000 m (Liao et al., 2021b). It is a novel berry with great potential for development. However, the resource investigation and research for many years showed that the mature period of wild *A. eriantha* was generally late, and the physiological mature period was concentrated from the end of October to early of November. The concentrated harvest time and short shelf life of fresh fruit would restrict the effective development of *A. eriantha* germplasm resources. Therefore, early maturity is of significance for enriching the mature period to meet market. Fortunately, our team found an early mature germplasm ‘Ganlv 2’ with excellent fruit traits at an altitude of 750 m in Nancheng County (Jiangxi Province, China) during the investigation of wild kiwifruit resources (Lang et al., 2016; Zhong et al., 2018). The germplasm matured more than 20 days earlier than the conventional cultivars or lines, which provided a valuable resource for germplasm innovation and cultivar improvement of *A. eriantha*. It also brought an opportunity to study the mechanism of early maturation in *A. eriantha*.

As we all know, the mature process is a complex process involving a series of physiological and biochemical pathways. At present, researchers at home and abroad use a single metabolic pathway to study the physiological basis of maturity differences. It is inevitable that certain metabolic pathways that play an important role are ignored. Therefore, the non-target metabolomics technology that uses unbiased analysis of samples can effectively solve this problem. It can efficiently identify the metabolites that are differentially expressed between cultivars with different maturity, and enrich the metabolites in various pathways to accurately detect the physiological basis for the formation of differences in maturity. Non-targeted metabonomics technology has been well applied in the analysis of different metabolites in grapes, citrus, and other species (Zhang et al., 2020; Alves et al., 2021; Cao et al., 2021). On the basis of metabolome, it is more purposeful to identify key regulatory genes in combination with transcriptome technology. There have been a lot of reports on the research of metabolomics and transcriptomics combined analysis (Savoi et al., 2016; Shen et al., 2020), while hardly reported in *A. eriantha*.

Therefore, in this study, the early mature germplasm ‘Ganlv 2’ and the excellent late mature germplasm ‘Ganlv 1’ found in the same region were used as materials to detect the fruit quality and endogenous hormones during the growth and development of the fruit. Meanwhile, through non-target metabonomics technology, we comprehensively identify the key metabolites formed by the early mature trait and mine the key regulatory genes for key metabolites by transcriptome. Finally, we use whole-genome resequencing technology to find the differential single nucleotide polymorphisms (SNP), insert or delete maker (InDel), structural variation (SV), and copy number variation (CNV) loci of key regulatory genes, and develop SNP loci that can be used for early mature germplasm identification. This study is expected to reveal the physiological basis and molecular mechanism of the formation of early mature trait of *A. eriantha*, and provide a theoretical basis for early mature germplasm innovation and cultivar improvement of *A. eriantha*.

## Materials and Methods

### Materials

**FIGURE 1:**
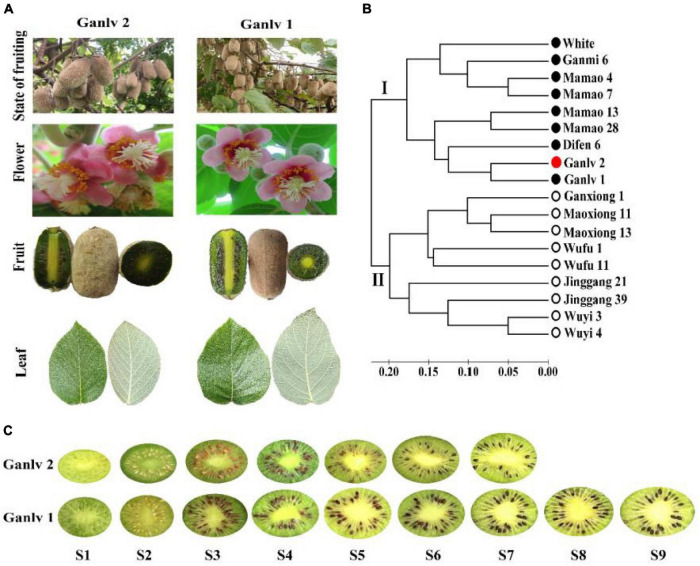
Test materials **(A)** ‘Ganlv 1’ and ‘Ganlv 2’ had similar botanical traits and flowering stages; **(B)** Simple sequence repetition (SSR) technology was used for genetic similarity coefficient analysis, and the genetic similarity coefficient value of test materials was less than 0.08, the bar at the bottom is the bar of genetic similarity coefficient, the solid circle represents the female germplasm, the hollow circle represents the male germplasm, and ‘Ganlv 2’ was marked in red; **(C)** Fruit developmental stages and sampling time points of ‘Ganlv 2’ and ‘Ganlv 1’, the early mature germplasm ‘Ganlv 2’ had reached the commercial harvested standard at S7.

The 5-year-old early mature *A. eriantha* germplasm, ‘Ganlv 2’, and late mature germplasm, ‘Ganlv 1’, were selected as the experimental materials, which planted in Shanwei kiwifruit germplasm resource nursery (E115°17′, N28°41′) in Fengxin County, Jiangxi Province, PR China. The parent materials of ‘Ganlv 1’ and ‘Ganlv 2’ were collected in the same place, had similar botanical traits and flowering stages, and the genetic similarity coefficient value was less than 0.08 (Figures 1A,B). A total of six vines, similar in size, bearing and receiving sunlight uniformly were used for the experiment and randomly divided into three groups (replicates) with two vines in each group. Fruit samples were collected from nine stages, which were as follows: 25 days after full bloom (DAF) (S1), 50 DAF (S2), 75 DAF (S3), 100 DAF (S4), 125 DAF (S5), 135 DAF (S6), 145 DAF (S7), 155 DAF (S8), and 165 DAF (S9) (Figure 1C). Sample handling refers to the previous study (Liao et al., 2021a), and stored at −80°C for later use. Three biological replicates were set for each period.

### Phenological Period Observation

According to our previous method (Liao et al., 2019b), we conducted a 3-year phenological observation of the test materials. It is very noteworthy that *A. eriantha* is a climacteric fruit, and the soluble solid content (SSC) of 6.5% is the general standard for fruit harvested of *A. eriantha*.

### Fruit Quality and Endogenous Hormone Content Assessment

The indicators of fruit quality determination include SSC, dry matter content (DM), soluble sugar content (TS), titratable acid content (TA), chlorophyll *a* content (Chl *a*), chlorophyll *b* content (Chl *b*), carotenoids content (Car), ascorbic acid content (AsA), starch content, and sugar components. Except for starch content was determined by iodine colorimetry (Xu et al., 1998), other indicators were determined by referring to our previous methods (Liao et al., 2020). In addition, according to previous studies (Chen et al., 1999), kiwifruit does not produce ethylene before harvest, so we only measured four key endogenous hormones: lower auxin (IAA), abscisic acid (ABA), zeatin (ZR), and gibberellin GA_3_. Endogenous hormones content was determined by high performance liquid chromatography.

### Non-target Metabolomics Analysis

In order to more comprehensively analyze the physiological basis of the formation of early maturity trait of *A. eriantha*, we commissioned Genedenovo Biotechnology Co., Ltd (Guangzhou, China) to perform non-targeted metabolomics sequencing. The extraction, sequencing, and bioinformatics analysis of metabolites were carried out according to routine procedures. LC-MS/MS analyses were performed using an UHPLC system (Vanquish, Thermo Fisher Scientific) with a UPLC BEH Amide column coupled to QE HFX mass spectrometer (Orbitrap MS, Thermo). The QE HFX mass spectrometer was used for its ability to acquire MS/MS spectra on information-dependent acquisition (IDA) mode in the control of the acquisition software (Xcalibur, Thermo). The raw data were converted to the mzXML format using ProteoWizard and processed with an in-house program, which was developed using R and based on XCMS, for peak detection, extraction, alignment, and integration. Then, an in-house MS2 database (BiotreeDB) was applied in metabolite annotation. Discriminant orthogonal partial least squares discriminant analysis (OPLS-DA) was used for model testing and screening of differential metabolites. This method can effectively reduce the complexity of the model and enhance the explanatory ability of the model without reducing the predictive ability of the model, so as to maximize the difference between groups.

### RNA Isolation, Library Preparation, and Transcriptome Sequencing

Total RNA was extracted by TRIzol reagent kit (TSINGKE Biotechnology Co., Ltd., Hunan, China) according to the protocol of the manufacturer. The quality of RNA was detected by an ultra-differential photometer and after, the mRNA with polyA tail was enriched with magnetic beads with OligodT. The obtained RNA was fragmented with the interrupted buffer, reverse transcription with random N6 primer, and then synthesized the cDNA two-strand to form double-stranded DNA. Then, the synthetic double-stranded DNA was filled in and the 5′ was phosphorylated. The 3′ forms a sticky end with an ‘A’ protruding, and then a blister linker with a protruding ‘T’ at the 3′ was connected. The ligation product was amplified by PCR with specific primers. The PCR product was heat-denatured into single-stranded, and then the single-stranded DNA was circularized with a bridge primer to obtain a single-stranded circular DNA library. Finally, it sequenced by BGISEQ-500 by BGI Medical Laboratory Co., Ltd (Wuhan, China). *A. eriantha* ‘White’ genome was as reference genome for data analysis (Tang et al., 2019). Sequencing data in our study was been uploaded to NCBI with the IDs PRJNA694809 and PRJNA694830.

### DNA Extraction and Resequencing Analysis

Leaf was used for DNA extraction, and DNA extraction and quality assessment was performed following the previously study (Liao et al., 2019a). We entrusted BGI Medical Laboratory Co., Ltd (Wuhan, PR China) to build the database and used BGISEQ-500/MGISEQ-2000 for sequencing. *A. eriantha* ‘White’ genome was also used as reference genome for data analysis. The sequencing process was carried out according to the conventional process. After filtering the raw data obtained by the instrument, GATK software was used to obtain SNP and InDel information, Breakdancer software to detect SV sites (Mckenna et al., 2010), and Control-FREEC software to find the CNV region between the sample and the reference genome (Boeva et al., 2012). Sequencing data in our study was uploaded to NCBI with the IDs PRJNA749653 and PRJNA749656.

### qRT-PCR Verification

We selected 25 key candidate genes for fluorescence quantitative analysis, and designed primers using Primer 5 based on the sequence information on the reference genome (Supplementary Table 1). The PCR reaction system preparation and reaction procedures refer to our previous methods (Liao et al., 2021a).

### Sanger Sequencing Verification

The content of DNA was diluted to 150 ng/μl and stored at −80°C. All SNP sites on key genes were for verification and were designed forward and reverse primers at the first and the last 150 bp of the SNP site by Primer 5 (Supplementary Table 2). The PCR reaction system also refers to our previous method (Liao et al., 2019a). Sequencing was done by TSINGKE Biotechnology Co., Ltd. (Hunan, China).

### Data Analysis

The experimental data were statistically analyzed and processed with Microsoft Excel 2016 and SPSS statistical package (IBM SPSS Statistics 22.0). A two-tailed Student’s *t*-test was used to detect differences at *p* ≤ 0.01. Origin 2018 was used to draw line and column charts and TBtools was used for gene co-expression network analysis (WGCNA) analysis.

According to previous research ideas (Guo et al., 2016), we selected S3, S5, S6, S7, S8, and S9 based on SSC for non-target metabolomic and transcriptomics analysis. The comparison group GL1 S3 vs. GL1 S5, GL1 S3 vs. GL1 S6, GL1 S3 vs. GL1 S7, GL1 S3 vs. GL1 S8, GL1 S3 vs. GL1 S9, GL2 S3 vs. GL2 S5, GL2 S3 vs. GL2 S6, and GL2 S3 vs. GL2 S7 were used for the identification and analysis of maturation-related key metabolites and genes, and GL2 S3 vs. GL2 S5, GL2 S3 vs. GL2 S6, GL2 S3 vs. GL2 S7, and GL2 S7 vs. GL1 S7 were used for the identification and analysis of key metabolites and genes related the formation of early mature trait.

## Results

### Comparison of Phenological Period

According to the distinctness, uniformity, and stability (DUS) test results, it can be seen that the early maturity trait of ‘Ganlv 2’ can be stably inherited, and the phenological period of the early maturity cultivar ‘Ganlv 2’ and the late maturity cultivar ‘Ganlv 1’ is only different in fruit maturing time, the fruit of ‘Ganlv 2’ maturated in early October, and the fruit of ‘Ganlv 1’ maturated in late October, about 20 days later than that of ‘Ganlv 2’ (Table 1).

**TABLE 1:** Comparison of phenological period between ‘Ganlv 2’ and ‘Ganlv 1’ (Month-Day).

**Stages**	**2017**		**2018**		**2019**	
	**‘Ganlv 2’**	**‘Ganlv 1’**	**‘Ganlv 2’**	**‘Ganlv 1’**	**‘Ganlv 2’**	**‘Ganlv 1’**
Stream injury	2-12	2-11	2-11	2-11	2-12	2-13
Stages in germination	2-27	2-28	2-26	2-26	2-28	2-26
Leaf spreading	3-9	3-9	3-8	3-7	3-8	3-8
Early flowering	5-2	5-1	5-1	5-3	5-1	5-1
Flowering	5-5	5-5	5-3	5-5	5-3	5-3
Terminal flowering	5-15	5-14	5-13	5-15	5-14	5-15
Fruit maturing	10-3	10-25	10-1	10-23	10-2	10-25
Leaf fall	11-28	11-27	11-27	11-27	11-28	11-27

### Fruit Quality and Endogenous Hormone Content Evaluation During Fruit Development

**FIGURE 2:**
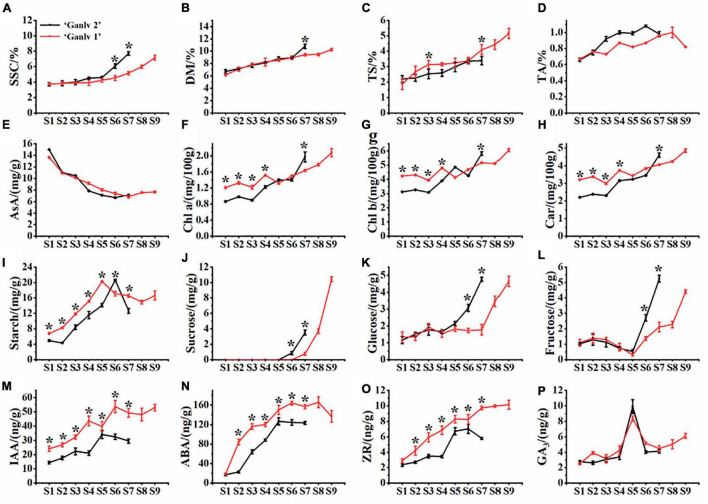
Dynamic change characteristics of inner quality[**(A)** soluble solids content, **(B)** dry matter, **(C)** soluble sugar content, **(D)** titratable acid content, **(E)** ascorbic acid content, **(F)** chlorophyll *a*, **(G)** chlorophyll *b*, **(H)** carotenoids, **(I)** starch, **(J)** sucrose, **(K)** glucose, and **(L)** fructose] and endogenous hormone content [**(M)** indole-3-acetic acid, **(N)** abscisic acid, **(O)** zeatin, and **(P)** gibberellin] of ‘Ganlv 2’ and ‘Ganlv 1’. *represents that there are significant differences between different cultivars at same period on same index, p 0.05. The red line represents ‘Ganlv 1’ and the black line represents ‘Ganlv 2’.

It can be seen from Figure 2 that ‘Ganlv 2’ reached the commercial harvest standard (SSC = 6.5%) at S6, while ‘Ganlv 1’ did not reach this harvest standard until S9 (Figure 2A). The change trend of DM and SSC was the same, but there was a significant difference only at S7 (Figure 2B). The TS content of ‘Ganlv 1’ was almost higher than that of ‘Ganlv 2’ in the whole growth period, but there was significant difference at S3 and S7 (Figure 2C). The TA content of ‘Ganlv 1’ and ‘Ganlv 2’ had the same trend of change, and both showed a decreasing trend at harvest time (Figure 2D). In AsA, ‘Ganlv 1’ and ‘Ganlv 2’ both showed a downward trend (Figure 2E). The changes of Chl *a*, Chl *b*, and Car of ‘Ganlv 1’ and ‘Ganlv 2’ were consistent throughout the growth period, the contents of Chl *a*, Chl *b*, and Car of ‘Ganlv 1’ were significantly higher than that of ‘Ganlv 2’ at S1–S3 period, but lower than that of ‘Ganlv 2’ at S7 (Figures 2F–H).

The starch content of ‘Ganlv 1’ gradually increased during S1–S5, and the peak value was at S5, and then showed a decreasing trend during S5–S8, the starch content at S8 decreased by 19.52% compared with that at S5 (Figure 2I). During S1–S5, the starch content of ‘Ganlv 2’ fruit was significantly lower than that of ‘Ganlv 1’. The glucose, fructose, sucrose, and starch contents of fruit were determined. The results showed that during the period of S1–S5, there was no significant difference in the glucose, sucrose, and fructose contents between the fruit of ‘Ganlv 1’ and ‘Ganlv 2’. However, in S6–S7 period, the glucose, sucrose, and fructose contents of ‘Ganlv 2’ were significantly higher than that of ‘Ganlv 1’. In addition, the fructose and glucose contents of the two cultivars at harvest stage were similar, but the sucrose content of ‘Ganlv 1’ was much higher than that of ‘Ganlv 2’ (Figures 2J–L).

The contents of IAA, ABA, GA_3_, and ZR were determined during the growth and development of fruit. The results showed that the IAA content of ‘Ganlv 2’ fruit was significantly lower than that of ‘Ganlv 1’ during the whole fruit development period, and the peak value of IAA content of ‘Ganlv 2’ fruit was at S5, and that of ‘Ganlv 1’ fruit was at S6 (Figure 2M). The ABA content of ‘Ganlv 2’ fruit was significantly lower than that of ‘Ganlv 1’ during S2–S7 fruit development, the peak ABA content of ‘Ganlv 2’ fruit was at S5, and that of ‘Ganlv 1’ fruit was at S6 (Figure 2N). The ZR content of ‘Ganlv 2’ fruit was significantly lower than that of ‘Ganlv 1’ during S2–S7 fruit development period, the peak ZR content of ‘Ganlv 2’ fruit was at S6, and that of ‘Ganlv 1’ fruit was at S7 (Figure 2O). There was no significant difference in GA_3_ content between ‘Ganlv 2’ and ‘Ganlv 1’ during fruit development, and the trend of change was the same (Figure 2P).

In order to further understand function of endogenous hormones during fruit development, the IAA, GA_3_, and ZR ratio of ABA was calculated, and as can be seen from the Supplementary Figure 1, in either fruit growth or development of the ‘Ganlv 2’ or ‘Ganlv 1’, IAA, GA_3_, and ZR ratio of ABA content have the same overall downward change trend.

### Identification of Key Metabolites Related to the Formation of Early Mature Trait

#### Identification of All Metabolites

We used a combination of positive ion mode (POS) and negative ion mode (NEG) to detect metabolites. There were differences in the total number of metabolites identified in the two ionization modes. POS and NEG modes identified 4,658 and 2,806 metabolites, respectively. Among them, the known metabolites were 1,378 and 440, and most of them were unknown metabolites.

#### Partial Least Squares-Discriminant Analysis

OPLS-DA can effectively reduce the complexity of the model and enhance the explanatory ability of the model without reducing the predictive ability of the model to maximize the difference between groups. As can be seen from Supplementary Figure 2, whether in POS or NEG mode, the differences between the comparison groups were more obvious, which can be used for later KEGG metabolic pathway analysis.

In order to verify the reliability of the OPLS-DA model, cross-validation and permutation tests were carried out on the model. The cross-validation results showed that no matter in the POS mode or the NEG mode, the reliability of the models of all comparison groups was high, the model predictive ability was good, and met requirements of the analysis (Supplementary Table 3). The permutation test results were consistent with the cross-validation results (Supplementary Figure 3).

#### Identification of Metabolites Related to Maturation of *A. eriantha*

**FIGURE 3:**
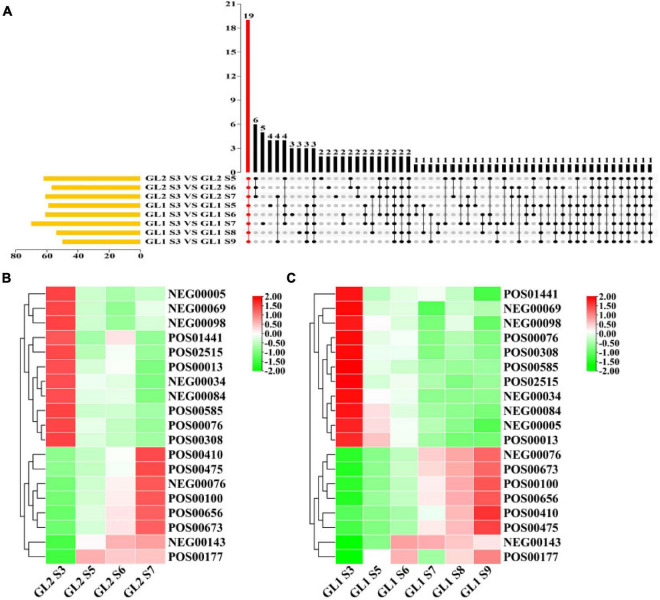
Upset analysis of maturation-related metabolites **(A)** and heat map of expression levels of metabolites on ‘Ganlv 2’ **(B)** and ‘Ganlv 1’ **(C)**. In **(A)** the yellow bar on the left represents the number of metabolites in the comparison group. The black dots on the right represent that these metabolites appear in the corresponding group, and the gray dots represent not. Metabolites present in all comparison groups were marked in red. In **(B,C)** the bar on the right represents the normalized coefficient range of the expression level. The redder the bar is, the higher the expression level, and the greener means the lower the expression level.

It can be seen from Figure 3A that there were 19 metabolites that were differentially expressed during fruit growth and development of ‘Ganlv 1’ and ‘Ganlv 2’ fruit. Among them, AsA was detected in both POS and NEG mode. To classify and analyze these metabolites, these metabolites were mainly concentrated in the related metabolic pathways such as stress resistance, sugar metabolism, aroma, phenolic acid, terpenes, and biofilm (Supplementary Table 4). These substances were closely related to fruit maturation. It can be seen from the heat map of the expression level of metabolites that the expression trend of metabolites was the same whether on ‘Ganlv 1’ or ‘Ganlv 2’. And it can be divided into two categories: the expression level increases with the growth period and the expression decreases with the growth period (Figures 3B,C).

#### Identification of Metabolites Related to Early Maturation of *A. eriantha*

**FIGURE 4:**
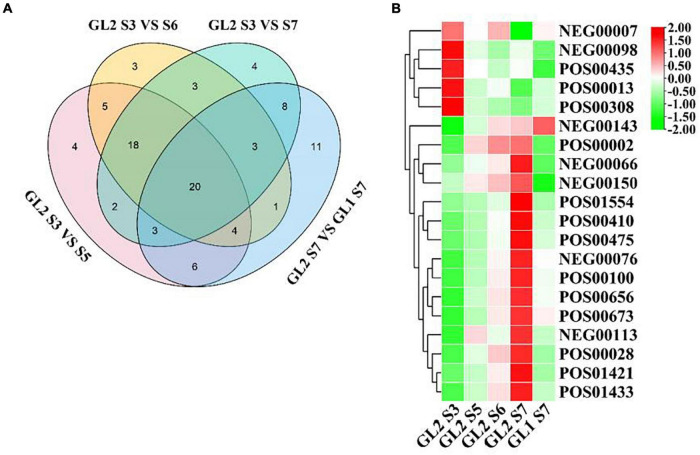
Venn diagram analysis of metabolites related to the formation of early mature trait **(A)** and heat map of expression levels of metabolites **(B)**. In **(A)** the number represents the number of metabolites contained in each area. In **(B)** the bar on the right represents the normalized coefficient range of the expression level. The redder the bar, the higher the expression level, and the greener bar, the lower the expression level.

According to the experimental design, 20 metabolites were identified as closely related to the formation of early mature trait of *A. eriantha* (Figure 4A). To classify and analyze these metabolites, KEGG analysis showed that these metabolites were mainly concentrated in anti-stress, sugar metabolism, aroma, phenolic acid, and other related metabolic pathways (Supplementary Table 5). It also can be seen from Figure 4B that in addition to NEG0007 metabolites, other metabolites can be divided into two categories on ‘Ganlv 2’: the expression level increases with the growth period and the expression level decreases with the growth period. When ‘Ganlv 2’ reached the harvest stage (GL2 S7), the content of most candidate metabolites in ‘Ganlv 2’ was significantly higher than in ‘Ganlv 1’ S7 period.

### Identification of Key Genes Related to the Formation of Early Mature Trait

#### Sequencing Quality and Trend Analysis of Gene Expression

The DNBSEQ platform was used to sequence 30 samples, as can be seen from Supplementary Table 6. Each sample produced an average of 6.47 GB of data. The average reference genome total mapping rate was 74.81% and reference gene set total mapping rate was 65.30%.

**FIGURE 5:**
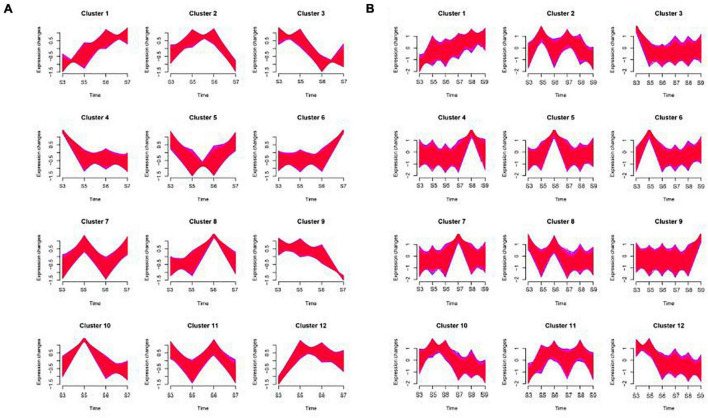
Time series analysis of genes on ‘Ganlv 2’ **(A)** and ‘Ganlv 1’ **(B)**. According to the trend of gene expression, genes with consistent expression patterns will be clustered into the same cluster. The red area was composed of multiple lines of gene expression line chart. The edges of the red area were framed with blue lines.

Some genes in the sample have similar expression patterns at different stages. It can be seen from the Figure 5 that both ‘Ganlv 1’ and ‘Ganlv 2’ can form 12 gene clusters, and the cluster 1, 2, 4, 6, 10, and 11 of ‘Ganlv 2’ was consistent with cluster 1, 5, 3, 9, 10, and 8 of ‘Ganlv 1’, respectively. The variation range of each gene cluster of ‘Ganlv 1’ was greater than that of ‘Ganlv 2’. On ‘Ganlv 2’, the expression patterns of cluster 6 was consistent with that of sucrose, glucose, and fructose, the expression patterns of cluster 8 was consistent with that of starch content, and the expression patterns of cluster 12 was consistent with that of IAA, ABA, and ZR content. On ‘Ganlv 1’, the expression patterns of cluster 9 was consistent with that of sucrose, glucose, and fructose. Expression patterns cluster 4 was consistent with that of starch content and expression patterns cluster 11 was consistent with that of IAA, ABA, and ZR content.

#### Identification of Genes Related to Maturation of *A. eriantha*

It can be seen from Supplementary Figure 4, there were only 2,433 DEGs were co-expressed in ‘Ganlv 2’ and ‘Ganlv 1’. Then Gene Ontology (GO) and KEGG analysis were carried out, the results showed that in GO analysis, the top five were partial membrane components, intrinsic membrane components, DNA-binding transcription factor activity, auxin activation signaling pathway, and cell response to auxin stimulation. In the KEGG analysis, 93, 200, 388, 1,142, and 138 DEGs were collected from the cellular process, environmental information processing, genetic information processing, metabolism, and organismal system, respectively, among which the global and overview maps metabolic process had the most DEGs (434).

#### Identification of Genes Related to Early Maturation of *A. eriantha*

Differentially expressed genes of S3 vs. S5, S3 vs. S6, S3 vs. S7 in ‘Ganlv 2’ and ‘Ganlv 2’ S7 vs. ‘Ganlv 1’ S7 were used to carry out digging DEGs related to the formation and regulation of early maturity traits. As can be seen from Supplementary Figure 5, a total of 3,132 DEGs were co-expressed in the above periods. In GO analysis, the top five were oxidoreductase activity, partial membrane components, intrinsic membrane components, membranes and partial membranes, and enriched 290, 967, 962, 1,047, and 992 DEGs, respectively. In the KEGG analysis, 124, 245, 460, 1,654, and 150 DEGs were collected from the cellular process, environmental information processing, genetic information processing, metabolism, and organismal system, respectively, among which the Global and overview maps metabolic process had the most DEGs (638).

#### Weighted Gene Co-expression Network Analysis

**FIGURE 6:**
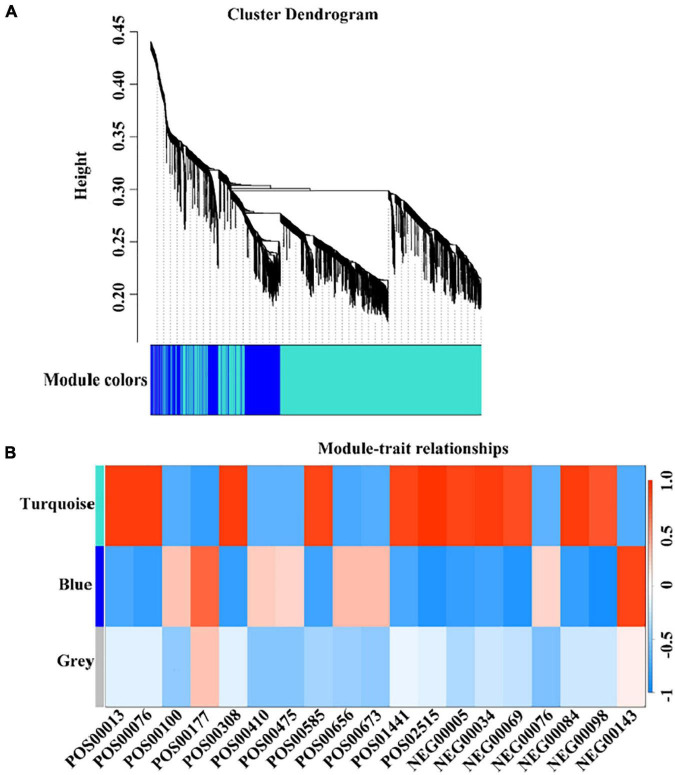
Weighted Gene Co-expression Network Analysis (WGCNA) of co-expressed DEGs. **(A)** The colors of each block indicate the correlation coefficient between the modules and the traits. **(B)** The bar on the right represents the range of correlation coefficients between modulus and traits. The redder means the more positive correlation, and the bluer means the more negative correlation.

Combining non-targeted metabolomics data and transcriptome gene expression data, we used TBtools to perform weighed gene co-expression network analysis (WGCNA). The results showed that 2,433 genes related to maturity can be divided into three color modules, namely turquoise module (1,770), blue module (561), and gray module (13) (Figure 6). At the same time, combined with the KEGG analysis of mature related genes, the metabolites and the color of the WGCNA analysis module were matched. A total of 73 genes were identified that were closely related to the metabolism of the corresponding metabolites. Among them, nine genes regulate multiple different metabolites (Supplementary Table 7). However, the key genes of aroma substances have not been identified, and it is very likely that there were other potential ways to regulate the content of metabolites.

**FIGURE 7:**
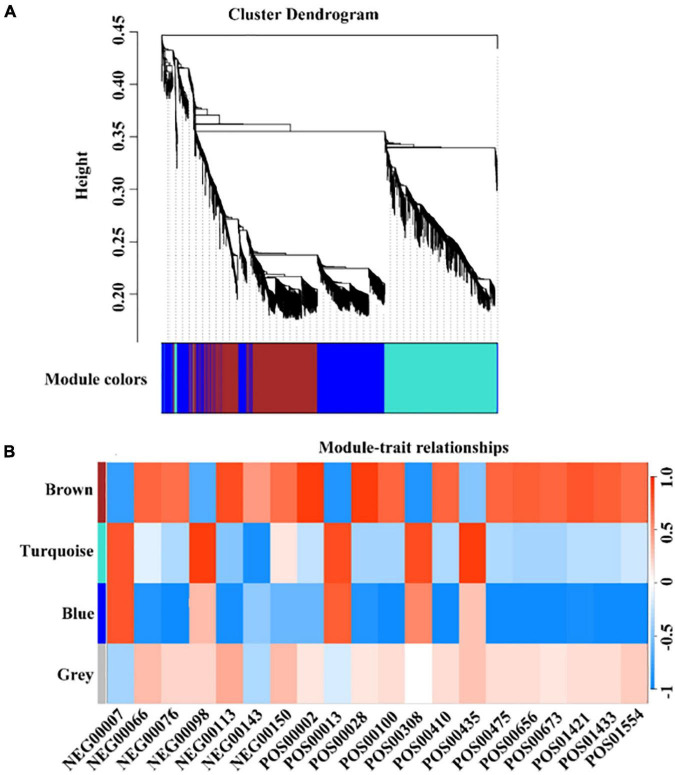
WGCNA analysis of early maturation-related genes and metabolites. **(A)** The colors of each block indicate the correlation coefficient between the modules and the traits. **(B)** The bar on the right represents the range of correlation coefficients between modulus and traits. The redder means the more positive correlation, and the bluer means the more negative correlation.

In terms of genes and metabolites related to the formation of early mature trait, WGCNA analysis results showed that 3,132 genes related to the formation of early mature trait can be divided into four color modules, namely, the brown module (931), the turquoise module (1,044), blue modules (1,026), and gray modules (3) (Figure 7). A total of 76 genes were identified that were closely related to the metabolism of corresponding metabolites, of which six genes regulate multiple different metabolites (Supplementary Table 8).

#### qRT-PCR Analysis

**FIGURE 8:**
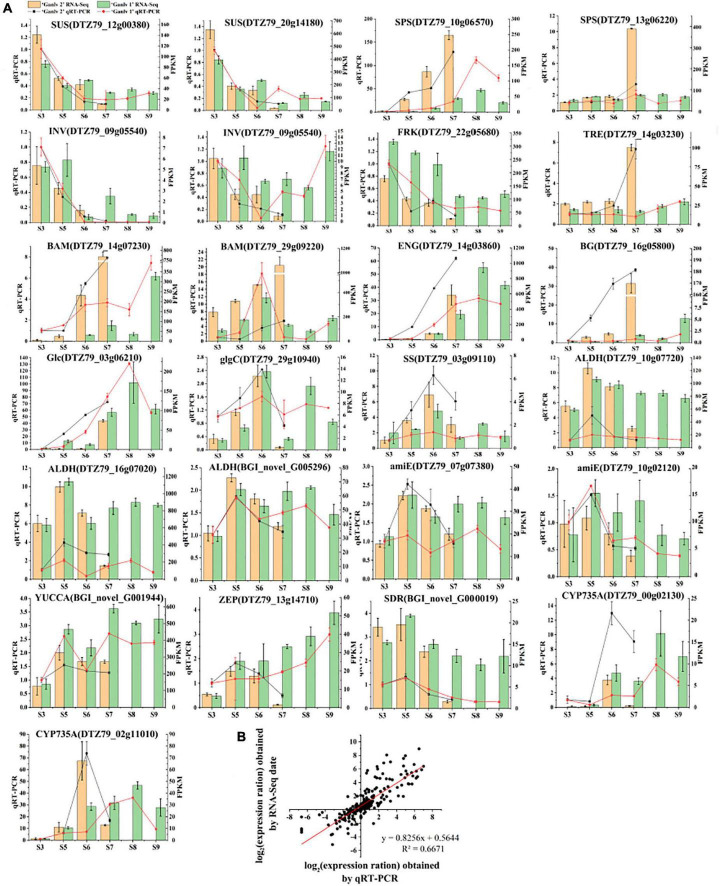
Analysis of gene expression of key candidate genes. Expression level by qRT-PCR and RNA-Seq **(A)**, the bar chart is RNA-seq data, yellow and green represent ‘Ganlv 2’ and ‘Ganlv 1’, respectively; the line chart was qRT-PCR data, black and red represent ‘Ganlv 2’ and ‘Ganlv 1’, respectively. Correlation analysis of RT-qPCR data and RNA-seq data **(B)**.

The relative expression of 26 key candidate genes was detected by qRT-PCR, and the quality of transcriptome sequencing was detected by correlation analysis. As can be seen from Figure 8, the transcriptome data and the RT-PCR data showed the same trend, and the correlation was 0.6671, which indicates that the transcriptome sequence data was reliable.

### Resequencing Analysis

#### Sequencing Quality

A total of 45.76 G of original sequencing data was generated by sequencing, and 44.65 G of high-quality and usable data was filtered by SOAPnuke software. The Q20 of ‘Ganlv 2’ and ‘Ganlv 1’ were both greater than 95%, Q30 was greater than 90%, the sequencing depth was 28.95 X and 32.46 X, respectively, and the matching ratio of the whole genome to Wat was 98.87% and 98.72%, respectively. The mapping rate to the whole genome of ‘white’ was 98.87% and 98.72%, respectively.

#### Difference Analysis With Reference Genome

Compared with the reference genome, ‘Ganlv 2’ and ‘Ganlv 1’ had 9,399,983 and 9,487,607 SNP sites, respectively. Among them, 9,393,020 and 9,306,154 were homozygous SNP sites, 5,839,927 and 5,785,585 mutation sites were converted type, and 3,622,964 and 3,588,924 mutation sites were transverted type.

There were 3,574,882 and 3,538,599 InDel sites on ‘Ganlv 2’ and ‘Ganlv 1’, respectively. Among them, 3,400,627 and 3,365,812 were homozygous InDel sites, 174,255 and 174,255 were heterozygous InDel sites, 1,841,505 and 1,822,968 InDel sites were insertion, and 1,733,377 and 1,715,631 InDel sites were deletion.

The insertion SV sites of ‘Ganlv 2’ and ‘Ganlv 1’ were 0, and the deletion SV sites were 16,506 and 5,405, respectively. Inversion SV sites were 17,705 and 10,970, respectively. There were 17,071 and 14,714 intra-chromosomal translocation SV loci, and 48,184 and 29,906 inter-chromosomal translocation SV sites, respectively.

The loss number and gain number of CNV on ‘Ganlv 2’ was more than that of ‘Ganlv 1’. They were 340 and 256, 190 and 62, respectively.

**FIGURE 9:**
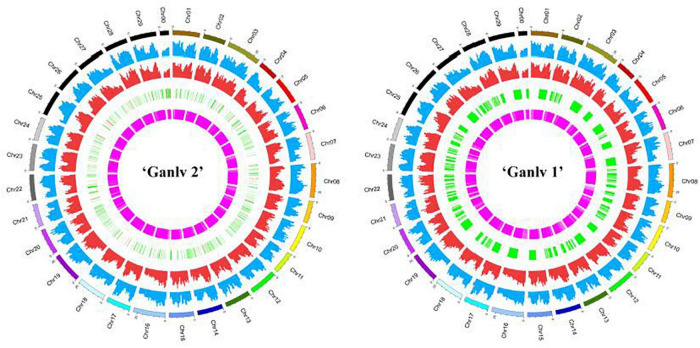
Circos of ‘Ganlv 2’ and ‘Ganlv 1’. The outermost circle represents the chromosome and its position. From the outside to the inside are single nucleotide polymorphism (SNP) frequency, Indel frequency, position and length of copy number variation (CNV), and position and length of structural variation (SV). In the circle where CNV is located, the red region represents chromosome copy number amplification, and the green region represents chromosome copy number deletion.

Finally, Circos software was used to draw the information of various mutation sites on chromosomes on a map (Figure 9). In addition, we annotated the gene information of SNP, InDel, SV, and CNV mutation sites and classified them according to whether the mutation sites would lead to changes in protein sequences. We found that the number of most types was close between ‘Ganlv 2’ and ‘Ganlv 1’ (Supplementary Table 9).

#### Identification of Key SNP Mutation Sites in Genes Related to the Formation of Early Mature Trait

We use the SnpEff software to classify the SNP sites on the early maturity germplasm ‘Ganlv 2’ according to the degree of influence of SNP on the protein structure. About 0.061% (10,028) of the SNP sites have a serious impact on the protein. According to the influence of the mutation site on protein coding, the mutation sites were classified. In order to ensure the accuracy of SNP sites and filter out low-quality sites, the sequencing depth was required to be ≥10X. There was a total of 42,969 genes with differential SNP sites on ‘Ganlv 2’, of which 7,331 SNP sites were key sites that seriously affect the structure of the protein. Combined with the analysis results of the identification of genes related to the formation of early mature trait, there were 24 SNP sites distributed on seventeen key candidate genes related to the formation of early mature trait (Supplementary Table 10). After final screening, 10 loci were completely different from the reference genome on ‘Ganlv 2’ and these 10 loci on ‘Ganlv 1’ were consistent with the reference genome. These 10 sites were distributed within eight genes (Table 2).

**TABLE 2:** Functional annotation of eight selected genes.

**Gene ID**	**Chromosome position**	**Star**	**End**	**Annotation**
DTZ79_02g02390	Chr02	1960389	1971794	pfkB-like carbohydrate kinase family protein
DTZ79_02g11010	Chr03	14372621	14376317	Cytochrome P450
DTZ79_03g06210	Chr03	6319452	6322290	Glucan endo-1,3-beta-glucosidase-like protein
DTZ79_03g09110	Chr03	9304895	9315519	Starch synthase
DTZ79_06g06870	Chr06	11792111	11797665	Trehalose-6-phosphate synthase
DTZ79_08g09930	Chr08	18490214	18501265	12-oxophytodienoate reductase-like protein
DTZ79_10g07840	Chr10	16467080	16477629	NAD kinase
DTZ79_27g04060	Chr27	4096070	4097397	Allene oxide cyclase

#### Sanger Sequencing Verification

Based on the resequencing data and SNP positions, ten pairs of primers were designed. The verification results of the first-generation sequencing were completely consistent with the re-sequencing screening results, which indicated that the 10 selected SNP loci could be used for the early identification of early mature trait of *A. eriantha* (Supplementary Table 2).

## Discussion

### Low Concentration of Endogenous Hormones Promote Starch Hydrolyzed to Sugars

Fruit maturation is a complex physiological process, which is accompanied by a series of biochemical processes such as sugar accumulation (Zhang Y.J. et al., 2014; Xu et al., 2020), acid reduction (Jiang et al., 2020), aroma production (Wei et al., 2016), pigment accumulation (Gan et al., 2019), flavonoid metabolism (Huang et al., 2020), and endogenous hormone change (Wang et al., 2016). The difference of fruit quality between early mature and late mature cultivar in our study was mainly reflected in the SSC, DM, and pigment contents. These results indicated that the metabolic pathways of starch and sugar and pigment involved in these indices were involved in the formation and regulation of the early mature trait of ‘Ganlv 2’. Studies on other fruit trees also showed that the starch and sugar metabolism, pigment synthesis, and hormone metabolism pathways are the main pathways that regulate fruit maturity (Xu, 2015), which are also the main research contents of the formation and regulation of early mature trait in *A. eriantha*.

In the mature process of *A. eriantha* fruit, the mature process can be divided into three important stages: cell division, fruit enlargement (mainly starch accumulation), and fruit ripening (Zhang H.Q. et al., 2014). Starch content will accumulate a lot in the early stage of fruit development and begin to decrease in the late stage of fruit development. Our findings were consistent with this and found that monosaccharides (fructose and glucose) were the main metabolisms in the early development stage of ‘Ganlv 1’ and ‘Ganlv 2’. Combined with the data of starch, we speculated that the formation of early mature character of *A. eriantha* was probably due to the faster rate of starch hydrolyzed to sucrose, which made the sucrose content significantly increase and reached the harvested standard.

**FIGURE 10:**
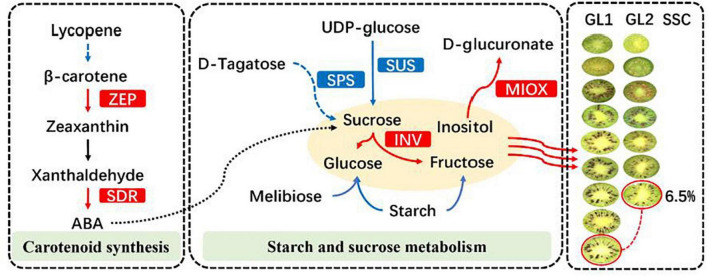
Some of the biological pathways involved in early mature in *A. eriantha*. The red box represents inhibition, and the blue box represents promotion.

At present, there have been many reports on the regulation of hormones in kiwifruit maturity, but mainly focused on the postharvest regulation. Most of the studies showed that the post-ripening softening process of kiwifruit was divided into two stages: the initial softening stage in the early stage and the rapid softening stage in the late stage. The main role of ethylene in the process was to accelerate the rapid softening stage of fruit softening process, but there was no obvious relationship between ethylene and the initial softening stage (Chen et al., 1999). Studies on *Actinidia deliciosa* have shown that maximum content of ABA was appeared at the early stage of fruit development and decreases with fruit maturity (Tao et al., 1994). The results of our study were contrary to this, which may be caused by the differences between genotypes, or it may be due to the earlier sampling time of previous studies and the analysis of samples taken between 0 and 100 days after pollination. Studies on the early maturity mutant of ‘Gannanzao’ navel orange showed that the late mature germplasm had higher IAA and GA_3_ content and lower ABA content (Chen et al., 2019). Our findings are similar with this, but there are also differences. Combined with the characters of hormonal and correlation analysis, we inferred that the formation of early maturity traits in *A. eriantha* was not caused by the difference in the proportion of hormones, but due to differences in hormone content. In addition, low concentration of IAA, ABA, and ZR can not only promote fruit development, but also promote fruit maturity by promoting starch hydrolysis to sugars (Figure 10).

### Sugar Metabolism Plays an Important Role in the Formation of Early Mature Trait

It is very interesting that the non-target metabolomics technology did not detect hormone-related metabolites. The main reason for this was that there were differences in the methods of identifying different metabolites. The results of hormone experiments were mainly the differences between early and late germplasms at the same period, while non-target metabolomics techniques were used to compare and analyze the metabolites in different developmental periods between early and late germplasms. This is the biggest reason why hormones were not detected. In addition, non-target metabolomics technology does not have the accuracy of target metabolomics data, and it is very likely that there was also a small part of low-level metabolites that have not been detected. The detection method also has a very important influence on the identification of metabolites (Lu et al., 2021). Those metabolites that participate in both the regulation of fruit mature and the formation of early mature trait were the most critical metabolites in this study.

In the sugar metabolism pathway, sucrose is an important sugar metabolite, and a large number of studies have reported that it is closely related to fruit maturing, and it is a direct promoter of fruit ripening in some sucrose-accumulating species (Zhou and Paull, 2001; Jia et al., 2013). In addition to sucrose, inositol, D-tagatose, and melibiose were also identified in this study. Among them, inositol could combine with IAA to form auxin conjugated substance involving in auxin transport and storage, this was related to our inferences about hormones. On the other hand, D-tagatose is a rare natural hexulose in nature. It belongs to a kind of rare sugar. It has various physiological effects such as inhibiting high blood sugar, improving intestinal flora, and preventing dental caries. Rare sugars refer to a type of monosaccharides that exist in nature but have very little content. In addition, D-tagatose is an epimer of fructose, and its sweetness is 92% of that of sucrose. It does not produce bad flavor and aftertaste. It is a good functional sweetener (Huang, 2008) and is widely used in food processing. At present, there were few research reports on the involvement of D-tagatose in fruit maturing, but from the analysis of the KEGG metabolic pathway analysis, D-tagatose can be converted into D-fructose-6 phosphate, thereby participating in the metabolism of sucrose. Therefore, it can be inferred that D-tagatose and sucrose have a synergistic role in regulating the maturing of *A. eriantha*. From the perspective of the expression of metabolites, the fruit of *A. eriantha* has a high content of natural D-tagatose, which can be an important raw material for the extraction of D-tagatose. Like D-tagatose, there were few reports on melibiose in fruit. After α-galactosidase acts on melibiose, it can be converted into galactose and glucose and participate in the metabolism of sucrose (Li, 2006).

Key genes related to sugar metabolism, such as sucrose synthase (SUS) (EC: 2.4.1.13) and sucrose phosphate synthase (SPS) (EC: 2.4.1.14) were mined out through transcriptome technology. Both are key enzymes involved in the process of sucrose synthesis and accumulation, and are mainly distributed in the cytoplasm. Among them, SUS is the only enzyme that enables sucrose to participate in various metabolic processes such as tissue construction, material storage, and plant cell metabolism, and it exists in most plants in the form of more than two isoenzymes (Moricuchi and Yamaki, 1988). However, SUS is a two-way enzyme, which makes SUS have a rich and diverse role in plant growth and development, such as regulating the decomposition and synthesis of sucrose (Zhou and Paull, 2001), regulating the production of uridine diphosphate glucose (Geigenberger and Stitt, 1993), and participating in cell differentiation (Ruan and Churey, 1998), improve plant stress resistance, etc (Jha and Dubey, 2004). Unlike SUS, the direction in which SPS synthesizes sucrose is irreversible and plays an important role in the sucrose accumulation pool, but its regulatory effects on sucrose accumulation in different plants and different species are quite different (Hubbard et al., 1991). At present, many studies have shown that the accumulation of sucrose during maturation is closely related to the increase of SUS and SPS activity (Stommel, 1992), and the results of this study were consistent with this. In the plant hormone synthesis pathway, a number of key candidate genes have been screened, such as the zeaxanthin cyclooxygenase gene (ZEP, DTZ79_13g14710) and the short-chain alcohol dehydrogenase/reductase gene (SDR, BGI_novel_G000019) involved in ABA biosynthesis. The expression of genes was close to the trend of changes in metabolites, which indicates that these genes were very likely to participate in the biosynthesis of plant endogenous hormones and play a key role.

Based on above, we infer that the formation of early mature trait of *A. eriantha* is mainly due to the significant metabolic differences in sugar metabolism. At S7 stage, the conversion efficiency of sucrose to fructose and glucose in the early mature germplasm fruit was reduced, the SSC was maintained and the rapid rising trend appeared so as to reach the harvested standard earlier than the late mature germplasm. As the formation of early mature trait, the gluconic acid and carnosic acid in the organic acid components were significantly reduced and accompanied by a large accumulation of volatile aroma substances precursor synthetic substances, which ultimately leads to the formation of the early mature trait of *A. eriantha* (Figure 10).

## Conclusion

In this study, we have made it clear that the early mature trait of ‘Ganlv 2’ can be stably inherited, and through characters of the fruit quality, endogenous hormone content and multi-omics analysis, we have determined the key metabolites and key genes related to the formation of early mature trait. Based on this, SNP sites that can be used to identify early mature trait were finally developed through resequencing technology. Therefore, we infer that the fruit of the early maturing germplasm ‘Ganlv 2’ has a low concentration of ABA, IAA, and ZR content, and under the action of these hormones, it promotes the accumulation of sucrose metabolism, which leads to the formation of early mature trait.

## Data Availability Statement

The datasets presented in this study can be found in online repositories. The names of the repository/repositories and accession number(s) can be found in the article/Supplementary Material.

## Author Contributions

GL: data analysis and writing-original draft preparation. YH and YL: collect fruit materials and fruit quality determination. QL and HW: metabolome data analysis. BY and DJ: real-time fluorescence quantification. MZ and CH: transcriptome data analysis. XX: writing-reviewing and editing. All authors read and approved the final manuscript.

## Conflict of Interest

The authors declare that the research was conducted in the absence of any commercial or financial relationships that could be construed as a potential conflict of interest.

## Publisher’s Note

All claims expressed in this article are solely those of the authors and do not necessarily represent those of their affiliated organizations, or those of the publisher, the editors and the reviewers. Any product that may be evaluated in this article, or claim that may be made by its manufacturer, is not guaranteed or endorsed by the publisher.

## Abbreviations

ABA: abscisic acid
AsA: ascorbic acid
CNV: copy number variation
DEGs: differentially expressed genes
GA: gibberellin
GO: Gene Ontology analysis
IAA: auxin
InDel: insert or delete maker
KEGG: Kyoto Encyclopedia of Genes and Genomes
NEG: negative ion mode
OPLS-DA: discriminant analysis of orthogonal least partial squares
POS: positive ion mode
SNP: single nucleotide polymorphisms
SV: structural variation
WGCNA: weighted correlation network analysis
ZEP: zeaxanthin epoxidase gene
ZR: zeatin.
