# Supplementary material for: Metabolome and Transcriptome Reveal Novel Formation Mechanism of Early Mature Trait in Kiwifruit (Actinidia eriantha)
Source: Front Plant Sci. 2021 Nov 19;12:760496. doi: 10.3389/fpls.2021.760496 (PMC8640357; doi:10.3389/fpls.2021.760496)
Supplement: Supplementary file 1 [file Table_1.docx]

Supplementary Table 1 Fluorescent quantitative primers.

| Gene name | Gene ID | Upstream primers (5’-3’) | Downstream primers (5’-3’) | Product size/bp |
| --- | --- | --- | --- | --- |
| *Actin* | | GTGCTCAGTGGTGGTTCAA | GACGCTGTATTTCCTCTCAG |  |
| *SUS* | DTZ79_12g00380 | GCTCACGCCTTGGAGAAAA | TCCTGGAAAGTGCTTGTGATTAT | 146 |
| *SUS* | DTZ79_20g14180 | CGCATTGAGAAGGTTTACGG | GCAACAAGGTTACCCTCGCTAT | 212 |
| *SPS* | DTZ79_10g06570 | CAAACAATCCGAAGTTCCTGAG | CCATTGTTGAGTGCCTTGAGA | 180 |
| *SPS* | DTZ79_13g06220 | CCCAAACACCACAAGCAGTC | TTGTCAAGAGCCCGATGTATGT | 188 |
| *INV* | DTZ79_03g04900 | CTGAAACGCCCAAGGAAACT | GCCACGGTGATGAGGAACA | 139 |
| *INV* | DTZ79_09g05540 | TGCTTGATGGTTGCTTCGTC | ACCCCTCATCGCAAAGACA | 101 |
| *FRK* | DTZ79_22g05680 | CGGAGCACTGCCTCGTATG | TCGTTGTCGCTGACCTTGAT | 122 |
| *TRE* | DTZ79_14g03230 | TTCGGGAGGTTTATTACTGGG | GAGGTTGGCTCCTATTCGTGT | 168 |
| *BAM* | DTZ79_14g07230 | TCAGTGCGGAGGAAATGTTG | CCTCTAAGCACTGGTAACGAATC | 162 |
| *BAM* | DTZ79_29g09220 | GTTCCGCTCGTTCACTCGT | GGGACTTGATAAGGCTTCGTTT | 192 |
| *ENG* | DTZ79_14g03860 | GAGGTGGGAGATGGGACAAC | GTCGGAAGGCTAAGGAGGC | 157 |
| *BG* | DTZ79_16g05800 | AGCATACAAGAATAAAGCCAAGC | GTTGGACCCAGGTTGGAGATA | 106 |
| *Glc* | DTZ79_03g06210 | ACAACGCAGCCACTTACAACC | GCCTCCCATCAGGGTGTAAC | 179 |
| *glgC* | DTZ79_29g10940 | ATCAATAGTGGCATCAACAAGGA | ATCAAACCGAAATCTGAAGCAC | 185 |
| *SS* | DTZ79_03g09110 | GTGAATGTTGTGAAAGGTGCG | ATTCAGGACACTCTTTCGGCTA | 138 |
| *ALDH* | DTZ79_10g07720 | CTCATCGGTCACGGCAAGA | GACACCAATGGCTCGTTTCC | 168 |
| *ALDH* | DTZ79_16g07020 | GAAACGGGGAAGATTGTGCT | ACGAGAACCCGCACAACAA | 180 |
| *amiE* | DTZ79_04g07060 | GCCCTGGTGGTTGGACATT | GCCCTGGTGGTTGGACATT | 128 |
| *amiE* | DTZ79_07g07380 | GGCAATCCCCACAGTTCCT | AGCAGCCGAGTGAGTCCGT | 193 |
| *amiE* | DTZ79_10g02120 | GATGGATACATTACTGGGTTTGG | CAGGTGTCCCATAGTGTTGATTT | 178 |
| *YUCCA* | BGI_novel_G001944 | GGAGGGCAAAAGCAGGAAG | CATCACTCCCACCTCGTTCC | 156 |
| *ALDH* | BGI_novel_G005296 | TCTCGGACTCGTTTCCATACA | AACAACCCACTTGCCCTCA | 71 |
| *ZEP* | DTZ79_13g14710 | AAGGTTTCAATGCCAGGTGC | GATACTGTTGCGACGGGGAT | 101 |
| *SDR* | BGI_novel_G000019 | CAGGCAAGGTGGCGGTAAT | GGCGAAAGAGCGAATGGAG | 149 |
| *CYP735A* | DTZ79_00g02130 | CCGAGTGGCAGGATAAGGTC | GGAAGAACTGATGCTGGTGGA | 145 |
| *CYP735A* | DTZ79_02g11010 | CCACTCCCTCACCATCTTACAG | CAATCTCCACGCAGTCTTTTCT | 173 |
