# Supplementary material for: Metabolome and Transcriptome Reveal Novel Formation Mechanism of Early Mature Trait in Kiwifruit (Actinidia eriantha)
Source: Front Plant Sci. 2021 Nov 19;12:760496. doi: 10.3389/fpls.2021.760496 (PMC8640357; doi:10.3389/fpls.2021.760496)
Supplement: Supplementary file 2 [file Table_2.docx]

Supplementary Table 2 Primers for SNP verification and results of Sanger sequencing.

| Code | Gene ID | Mutation site | Primer sequence | Sanger sequencing verification results | |
| --- | --- | --- | --- | --- | --- |
|  |  |  |  | GL2 | GL1 |
| 1 | DTZ79_02g02390 | 1965178 | F: CCGTTAAGCAATTGACTTAT  R: CACTATTCAGTTGACATCAT | T/T | C/C |
| 2 | DTZ79_02g02390 | 1965185 | F: CCGTTAAGCAATTGACTTAT  R: CACTATTCAGTTGACATCAT | G/G | T/T |
| 3 | DTZ79_02g11010 | 14374736 | F: GAACAATGGATCCTACTA  R: TGCTATGTGGGTTCACCA | A/A | G/G |
| 4 | DTZ79_03g06210 | 6321249 | F: GTATGATGAGAATCAAAAAGG  R: GCCTATCCACGGAAGCATCTT | C/C | T/T |
| 5 | DTZ79_03g09110 | 9312488 | F: CGATGATACTGAATGGAACCC  R: TCAATGATGCAACTTAATCCTCA | G/G | A/A |
| 6 | DTZ79_06g06870 | 11793325 | F: CAATTTCTATAGTTTTGTTAGTA  R: CTTTGGATCAAGCCAGGCTAAGG | T/T | C/C |
| 7 | DTZ79_08g09930 | 18499599 | F: CATGTTATGTCCAAGTCGCCC  R: TGCCAATAAGAGAGTGCAGTAC | T/T | C/C |
| 8 | DTZ79_10g07840 | 16471213 | F: TCTCTTGGTGATACAAGAAAG  R: CATACCATTTTAACTTCTGT | T/T | C/C |
| 9 | DTZ79_10g07840 | 16474717 | F: TCGCTGATGCACACATATCTC  R: GAAACTACAGATTCTGAGGTTG | T/T | A/A |
| 10 | DTZ79_27g04060 | 4096161 | F: GTGTGTATGAGATCAACGAGC  R: CCCGAGTCTACAACTTACCAA | C/C | G/G |
