# Supplementary material for: Metabolome and Transcriptome Reveal Novel Formation Mechanism of Early Mature Trait in Kiwifruit (Actinidia eriantha)
Source: Front Plant Sci. 2021 Nov 19;12:760496. doi: 10.3389/fpls.2021.760496 (PMC8640357; doi:10.3389/fpls.2021.760496)
Supplement: Supplementary file 3 [file Table_3.docx]

Supplementary Table 3 OPLS-DA model cross-validation results. R2X and R2Y represent the interpretation rate of the X and Y matrices of the built model, respectively. Q2 represents the predictive ability of the model, Q2>0.5 indicates that the model predicts better, and Q2>0.9 is an excellent model.

| Comparison group | R2X(cum) | | R2Y(cum) | | Q2(cum) | |
| --- | --- | --- | --- | --- | --- | --- |
|  | POS | NEG | POS | NEG | POS | NEG |
| GL2 S3 vs GL2 S5 | 0.468 | 0.358 | 0.991 | 0.982 | 0.925 | 0.868 |
| GL2 S3 vs GL2 S6 | 0.516 | 0.401 | 0.996 | 0.996 | 0.922 | 0.882 |
| GL2 S3 vs GL2 S7 | 0.601 | 0.505 | 0.997 | 0.992 | 0.977 | 0.903 |
| GL1 S3 vs GL1 S5 | 0.349 | 0.360 | 0.996 | 0.979 | 0.879 | 0.752 |
| GL1 S3 vs GL1 S6 | 0.388 | 0.427 | 0.991 | 0.929 | 0.846 | 0.670 |
| GL1 S3 vs GL1 S7 | 0.482 | 0.455 | 0.994 | 0.969 | 0.946 | 0.820 |
| GL1 S3 vs GL1 S8 | 0.426 | 0.480 | 0.997 | 0.957 | 0.941 | 0.834 |
| GL1 S3 vs GL1 S9 | 0.462 | 0.520 | 0.997 | 0.955 | 0.955 | 0.847 |
| GL1 S7 vs GL2 S7 | 0.534 | 0.421 | 0.995 | 0.957 | 0.954 | 0.686 |
