# Supplementary material for: Metabolome and Transcriptome Reveal Novel Formation Mechanism of Early Mature Trait in Kiwifruit (Actinidia eriantha)
Source: Front Plant Sci. 2021 Nov 19;12:760496. doi: 10.3389/fpls.2021.760496 (PMC8640357; doi:10.3389/fpls.2021.760496)
Supplement: Supplementary file 4 [file Table_4.docx]

Supplementary Table 4 Annotation of differential metabolites related mature.

| Code | Metabolite name | ID | KEGG pathway of metabolites | | Remarks |
| --- | --- | --- | --- | --- | --- |
| 1 | Stearic acid | NEG00005 | ko01040 | Biosynthesis of unsaturated fatty acids | Anti-cold damage substance, one of the main components of fatty acid |
| 2 | 13-OxoODE | NEG00034 | ko00591 | Linoleic acid metabolism | Precursor substance for synthesis of butanol dehydrogenase |
| 3 | Ascorbic acid | POS00076 | ko00053 | Ascorbate and aldarate metabolism | Antioxidant, commonly known as vitamin C |
| 4 |  | NEG00069 |  |  |  |
| 5 | Sucrose | NEG00076 | ko00500 | Starch and sucrose metabolism | Key energy and metabolites |
| 6 | Methyl jasmonate | NEG00084 | ko00592 | alpha-Linolenic acid metabolism | Antioxidant substances, often used for postharvest storage |
| 7 | Gluconic acid | NEG00098 | ko00030 | Pentose phosphate pathway | One of the main components of organic acids |
| 8 | L-Aspartic acid | NEG00143 | ko00300 | Lysine biosynthesis | Amino acid substance, one of the main components of organic acid |
| 9 | Carnosic acid | POS00013 | ko00904 | Diterpenoid biosynthesis | Phenolic acid compound, antioxidant substance |
| 10 | 2-Furancarboxaldehyde | POS00100 | ko01120 | Microbial metabolism in diverse environments | Aroma substances |
| 11 | Glutathione | POS00177 | ko00480 | Glutathione metabolism | Antioxidant substances |
| 12 | 4-Carboxymethylenebut-2-en-4-olide | POS00308 | ko01120 | Microbial metabolism in diverse environments | Catechol degrading substance, a dienolactone hydrolase that is active on both cis and trans dienolactones |
| 13 | D-Tagatose | POS00410 | ko00052 | Galactose metabolism | The isomer of D-galactose, the sweetness is similar to sucrose |
| 14 | 5-Hydroxymethyl-2-furancarboxaldehyde | POS00475 | ko01120 | Microbial metabolism in diverse environments | Aroma substances |
| 15 | Phosphorylcholine | POS00585 | ko00564 | Glycerophospholipid metabolism | One of the important components of biofilm |
| 16 | 1,3,5-Trihydroxybenzene | POS00656 | ko01120 | Microbial metabolism in diverse environments | Phenolic acids, often mixed with monoterpenes, sesquiterpenes, and diterpenes |
| 17 | Melibiose | POS00673 | ko00052 | Galactose metabolism | Natural disaccharides widely found in plant tissues |
| 18 | 4-Hydroxybutanoate | POS01441 | ko00650 | Butanoate metabolism | Aroma substances |
| 19 | 4-Hydroxylamino-2,6-dinitrotoluene | POS02515 | ko01120 | Microbial metabolism in diverse environments | Main metabolites of trinitrotoluene |
