# Supplementary material for: Metabolome and Transcriptome Reveal Novel Formation Mechanism of Early Mature Trait in Kiwifruit (Actinidia eriantha)
Source: Front Plant Sci. 2021 Nov 19;12:760496. doi: 10.3389/fpls.2021.760496 (PMC8640357; doi:10.3389/fpls.2021.760496)
Supplement: Supplementary file 5 [file Table_5.docx]

Supplementary Table 5 Annotation of differential metabolites related early mature.

| Code | Metabolite name | ID | KEGG pathway of metabolites | | Remarks |
| --- | --- | --- | --- | --- | --- |
| 1 | Alpha-Linolenic acid | NEG00007 | ko00592 | alpha-Linolenic acid metabolism | Unsaturated fatty acids with important physiological functions |
| 2 | myo-Inositol | NEG00066 | ko00053 | Ascorbate and aldarate metabolism | One of the main sugar components and an important precursor for the synthesis of ascorbic acid |
| 3 | Sucrose | NEG00076 | ko00500 | Starch and sucrose metabolism | Key energy and metabolites |
| 4 | Gluconic acid | NEG00098 | ko00030 | Pentose phosphate pathway | One of the main components of organic acids |
| 5 | L-Lactic acid | NEG00113 | ko00051 | Fructose and mannose metabolism | One of the main components of organic acids |
| 6 | L-Aspartic acid | NEG00143 | ko00300 | Lysine biosynthesis | One of the main components of organic acids |
| 7 | 3-Methylxanthine | NEG00150 | ko00232 | Caffeine metabolism | An important intermediate for the synthesis of purine derivatives |
| 8 | D-Proline | POS00002 | ko00330 | Arginine and proline metabolism | Amino acid substances, often used as resistance identification indicators |
| 9 | Carnosic acid | POS00013 | ko00904 | Diterpenoid biosynthesis | Phenolic acid compound, antioxidant substance |
| 10 | L-Serine | POS00028 | ko00260 | Glycine, serine and threonine metabolism | Amino acid substance |
| 11 | 2-Furancarboxaldehyde | POS00100 | ko01120 | Microbial metabolism in diverse environments | Aroma substances |
| 12 | 4-Carboxymethylenebut-2-en-4-olide | POS00308 | ko01120 | Microbial metabolism in diverse environments | Catechol degrading substance, a dienolactone hydrolase that is active on both cis and trans dienolactones |
| 13 | D-Tagatose | POS00410 | ko00052 | Galactose metabolism | The isomer of D-galactose, the sweetness is similar to sucrose |
| 14 | 3-Succinoylpyridine | POS00435 | ko00760 | Nicotinate and nicotinamide metabolism | Aroma substances |
| 15 | 5-Hydroxymethyl-2-furancarboxaldehyde | POS00475 | ko01120 | Microbial metabolism in diverse environments | Aroma substances |
| 16 | 1,3,5-Trihydroxybenzene | POS00656 | ko01120 | Microbial metabolism in diverse environments | Phenolic acids, often mixed with monoterpenes, sesquiterpenes, and diterpenes |
| 17 | Melibiose | POS00673 | ko00052 | Galactose metabolism | Natural disaccharides widely found in plant tissues |
| 18 | Oxamate | POS01421 | ko00230 | Purine metabolism | Often used for postharvest storage and preservation |
| 19 | Phosphoguanidinoacetate | POS01433 | ko00330 | Arginine and proline metabolism | One of the metabolites of guanidinoacetic acid, involved in antioxidant and sugar metabolism |
| 20 |  | POS01554 |  |  |  |
