# Supplementary material for: Metabolome and Transcriptome Reveal Novel Formation Mechanism of Early Mature Trait in Kiwifruit (Actinidia eriantha)
Source: Front Plant Sci. 2021 Nov 19;12:760496. doi: 10.3389/fpls.2021.760496 (PMC8640357; doi:10.3389/fpls.2021.760496)
Supplement: Supplementary file 6 [file Table_6.docx]

Supplementary Table 6 Quality analysis of RNA-Seq sequencing.

| Samples | Total raw reads/M | Total clean reads/M | Total clean bases/Gb | Q20/% | Q30/% | Reference genome  total mapping/% | Reference gene set total mapping /% |
| --- | --- | --- | --- | --- | --- | --- | --- |
| ‘Ganlv 2’ S3-1 | 43.82 | 43.28 | 6.49 | 98.12 | 94.82 | 75.03 | 64.95 |
| ‘Ganlv 2’ S3-2 | 43.82 | 43.25 | 6.49 | 97.86 | 94.11 | 75.11 | 67.06 |
| ‘Ganlv 2’ S3-3 | 43.82 | 43.26 | 6.49 | 97.88 | 94.20 | 74.95 | 66.14 |
| ‘Ganlv 2’ S5-1 | 43.82 | 43.34 | 6.50 | 98.06 | 94.62 | 73.68 | 64.25 |
| ‘Ganlv 2’ S5-2 | 43.82 | 43.27 | 6.49 | 97.91 | 94.25 | 74.61 | 66.24 |
| ‘Ganlv 2’ S5-3 | 43.82 | 43.25 | 6.49 | 97.86 | 94.12 | 74.28 | 65.93 |
| ‘Ganlv 2’ S6-1 | 43.82 | 43.25 | 6.49 | 97.75 | 93.84 | 74.41 | 65.60 |
| ‘Ganlv 2’ S6-2 | 43.82 | 43.25 | 6.49 | 97.75 | 93.84 | 74.58 | 66.44 |
| ‘Ganlv 2’ S6-3 | 43.82 | 42.97 | 6.45 | 97.45 | 93.18 | 74.19 | 65.31 |
| ‘Ganlv 2’ S7-1 | 43.82 | 43.33 | 6.50 | 97.94 | 94.26 | 74.43 | 64.94 |
| ‘Ganlv 2’ S7-2 | 43.82 | 43.34 | 6.50 | 97.94 | 94.28 | 74.28 | 65.01 |
| ‘Ganlv 2’ S7-3 | 43.82 | 43.32 | 6.50 | 98.02 | 94.47 | 74.64 | 64.96 |
| ‘Ganlv 1’ S3-1 | 43.82 | 42.92 | 6.44 | 97.62 | 93.65 | 74.78 | 66.62 |
| ‘Ganlv 1’ S3-2 | 43.82 | 42.97 | 6.44 | 97.53 | 93.43 | 74.18 | 65.86 |
| ‘Ganlv 1’ S3-3 | 43.82 | 42.88 | 6.43 | 97.66 | 93.77 | 74.25 | 64.69 |
| ‘Ganlv 1’ S5-1 | 43.82 | 43.25 | 6.49 | 97.93 | 94.29 | 74.82 | 65.90 |
| ‘Ganlv 1’ S5-2 | 43.82 | 43.28 | 6.49 | 97.9 | 94.23 | 74.47 | 64.94 |
| ‘Ganlv 1’ S5-3 | 43.82 | 43.23 | 6.48 | 97.99 | 94.46 | 75.01 | 65.81 |
| ‘Ganlv 1’ S6-1 | 43.82 | 43.28 | 6.49 | 97.90 | 94.23 | 74.89 | 65.71 |
| ‘Ganlv 1’ S6-2 | 43.82 | 42.92 | 6.44 | 97.56 | 93.50 | 74.88 | 65.10 |
| ‘Ganlv 1’ S6-3 | 43.82 | 42.96 | 6.44 | 97.58 | 93.56 | 73.86 | 64.52 |
| ‘Ganlv 1’ S7-1 | 43.82 | 43.23 | 6.48 | 97.90 | 94.22 | 76.36 | 66.22 |
| ‘Ganlv 1’ S7-2 | 43.82 | 42.91 | 6.44 | 97.70 | 93.84 | 75.95 | 65.59 |
| ‘Ganlv 1’ S7-3 | 43.82 | 42.93 | 6.44 | 97.50 | 93.36 | 75.09 | 65.35 |
| ‘Ganlv 1’ S8-1 | 43.82 | 42.98 | 6.45 | 97.45 | 93.19 | 75.04 | 64.54 |
| ‘Ganlv 1’ S8-2 | 43.82 | 42.97 | 6.45 | 97.46 | 93.19 | 75.19 | 64.84 |
| ‘Ganlv 1’ S8-3 | 43.82 | 43.30 | 6.50 | 97.78 | 93.91 | 75.19 | 64.84 |
| ‘Ganlv 1’ S9-1 | 43.82 | 42.94 | 6.44 | 97.59 | 93.57 | 74.54 | 63.44 |
| ‘Ganlv 1’ S9-2 | 43.82 | 43.18 | 6.48 | 97.96 | 94.40 | 75.73 | 64.68 |
| ‘Ganlv 1’ S9-3 | 43.82 | 43.27 | 6.49 | 97.88 | 94.19 | 75.97 | 63.59 |
| Mean | 43.82 | 43.15 | 6.47 | 97.78 | 93.97 | 74.81 | 65.30 |
