# Supplementary material for: Metabolome and Transcriptome Reveal Novel Formation Mechanism of Early Mature Trait in Kiwifruit (Actinidia eriantha)
Source: Front Plant Sci. 2021 Nov 19;12:760496. doi: 10.3389/fpls.2021.760496 (PMC8640357; doi:10.3389/fpls.2021.760496)
Supplement: Supplementary file 7 [file Table_7.docx]

Supplementary Table 7 Correlation analysis of maturation-related metabolites and genes.

| Metabolite ID | Metabolite name | Pathway ID | Pathway Name | Number of genes in the pathway | WGCNA | |
| --- | --- | --- | --- | --- | --- | --- |
|  |  |  |  |  | Module colors | Number of genes |
| NEG00098 | Gluconic acid | ko00030 | Pentose phosphate pathway | 8 | Turquoise | 3 |
| POS00410 | D-tagatose | ko00052 | Galactose metabolism | 21 | Blue | 7 |
| POS00673 | Melibiose |  |  |  | Blue |  |
| NEG00069 | Ascorbic acid | ko00053 | Ascorbate and aldarate metabolism | 17 | Turquoise | 9 |
| POS00076 |  |  |  |  | Turquoise |  |
| NEG00143 | L-Aspartic Acid | ko00300 | Lysine biosynthesis | 1 | Blue | 0 |
| POS00177 | Glutathione | ko00480 | Glutathione metabolism | 12 | Blue | 8 |
| NEG00076 | Sucrose | ko00500 | Starch and sucrose metabolism | 55 | Blue | 13 |
| POS00585 | Acylglycerophosphocholine | ko00564 | Glycerophospholipid metabolism | 16 | Turquoise | 12 |
| NEG00034 | 13-oxooctadecadienoic acid | ko00591 | Linoleic acid metabolism | 6 | Turquoise | 6 |
| NEG00084 | Methyl Jasmonate | ko00592 | alpha-Linolenic acid metabolism | 15 | Turquoise | 11 |
| POS01441 | 4-hydroxybutyric acid | ko00650 | Butanoate metabolism | 7 | Turquoise | 6 |
| POS00013 | Carnosic acid | ko00904 | Diterpenoid biosynthesis | 4 | Turquoise | 3 |
| NEG00005 | Stearic acid | ko01040 | Biosynthesis of unsaturated fatty acids | 7 | Turquoise | 4 |
| POS00100 | 2-furaldehyde | ko01120 | Microbial metabolism in diverse environments | 0 | - | - |
| POS00308 | Trans dilactone |  |  |  |  |  |
| POS00475 | 5-hydroxymethyl-2-furaldehyde |  |  |  |  |  |
| POS00656 | 1,3,5-Trihydroxybenzene |  |  |  |  |  |
| POS02515 | 4-hydroxyamino-2,6-dinitrotoluene |  |  |  |  |  |
