# Supplementary material for: Metabolome and Transcriptome Reveal Novel Formation Mechanism of Early Mature Trait in Kiwifruit (Actinidia eriantha)
Source: Front Plant Sci. 2021 Nov 19;12:760496. doi: 10.3389/fpls.2021.760496 (PMC8640357; doi:10.3389/fpls.2021.760496)
Supplement: Supplementary file 8 [file Table_8.docx]

Supplementary Table 8 Correlation analysis of early maturation-related metabolites and genes.

| Metabolite ID | Metabolite name | Pathway ID | Pathway Name | Number of genes in the pathway | WGCNA | |
| --- | --- | --- | --- | --- | --- | --- |
|  |  |  |  |  | Module colors | Number of genes |
| NEG00098 | Gluconic acid | ko00030 | Pentose phosphate pathway | 20 | Turquoise | 3 |
| NEG00113 | L-lactic acid | ko00051 | Fructose and mannose metabolism | 21 | Brown | 6 |
| POS00410 | D-tagatose | ko00052 | Galactose metabolism | 29 | Brown | 6 |
| POS00673 | Melibiose |  |  |  | Brown |  |
| NEG00066 | Inositol | ko00053 | Ascorbate and aldarate metabolism | 20 | Brown | 5 |
| POS01421 | Oxalate | ko00230 | Purine metabolism | 20 | Brown | 3 |
| NEG00150 | 3-methylxanthine | ko00232 | Caffeine metabolism | 4 | Brown | 0 |
| POS00028 | L-serine | ko00260 | Glycine, serine and threonine metabolism | 14 | Brown | 4 |
| NEG00143 | L-Aspartic Acid | ko00300 | Lysine biosynthesis | 3 | Brown | 0 |
| POS00002 | D-Proline | ko00330 | Arginine and proline metabolism | 24 | Brown | 2 |
| POS01433 | Guanidine Phosphate Acetate |  |  |  | Brown |  |
| POS01554 | Guanidine Phosphate Acetate |  |  |  | Brown |  |
| NEG00076 | Sucrose | ko00500 | Starch and sucrose metabolism | 84 | Brown | 20 |
| NEG00007 | Alpha-linolenic acid | ko00592 | alpha-Linolenic acid metabolism | 29 | Turquoise and blue | 26 |
| POS00435 | 3-succinylpyridine | ko00760 | Nicotinate and nicotinamide metabolism | 5 | Turquoise | 4 |
| POS00013 | Carnosic acid | ko00904 | Diterpenoid biosynthesis | 8 | Turquoise and blue | 3 |
| POS00100 | 2-furaldehyde | ko01120 | Microbial metabolism in diverse environments | 0 | - | - |
| POS00308 | Trans dilactone |  |  |  |  |  |
| POS00475 | 5-hydroxymethyl-2-furaldehyde |  |  |  |  |  |
| POS00656 | 1,3,5-Trihydroxybenzene |  |  |  |  |  |
