# Supplementary material for: Metabolome and Transcriptome Reveal Novel Formation Mechanism of Early Mature Trait in Kiwifruit (Actinidia eriantha)
Source: Front Plant Sci. 2021 Nov 19;12:760496. doi: 10.3389/fpls.2021.760496 (PMC8640357; doi:10.3389/fpls.2021.760496)
Supplement: Supplementary file 9 [file Table_9.docx]

Supplementary Table 9 Statistics of annotation.

| Types | SNP | | InDel | | SV | | CNV | |
| --- | --- | --- | --- | --- | --- | --- | --- | --- |
| Cultivars | ‘Ganlv 2’ | ‘Ganlv 1’ | ‘Ganlv 2’ | ‘Ganlv 1’ | ‘Ganlv 2’ | ‘Ganlv 1’ | ‘Ganlv 2’ | ‘Ganlv 1’ |
| Upstream | 645,214 | 629,195 | 306,885 | 300,203 | 7,552 | 4,519 | 0 | 3 |
| Stop gain | 5,506 | 5,523 | 2,023 | 2,085 | / | / | / | / |
| Stop loss | 882 | 885 | / | / | / | / | / | / |
| Frameshift deletion | / | / | 419 | 416 | / | / | / | / |
| Synonymous | 218,083 | 221,015 | / | / | / | / | / | / |
| Frameshift insertion | / | / | 34,384 | 34,435 | / | / | / | / |
| Non-synonymous | 257,436 | 260,329 | / | / | / | / | / | / |
| Non-frameshift deletion | / | / | 38,564 | 38,767 | / | / | / | / |
| Intronic | 2,139,511 | 2,114,509 | 7,799 | 7,916 | 38,211 | 25,107 | 0 | 0 |
| Exonic | / | / | / | / | 41,022 | 23,681 | 524 | 303 |
| Splicing | 3,382 | 3,376 | 5,454 | 5,396 | 252 | 155 | 0 | 0 |
| Downstream | 551,552 | 538,582 | 839,906 | 827,713 | 7,874 | 5,020 | 0 | 0 |
| upstream/downstream | 30,569 | 29,812 | 2,576 | 2,548 | 534 | 363 | 0 | 0 |
| UTR5 | 0 | 0 | 229,865 | 225,767 | 0 | 0 | 0 | 0 |
| UTR3 | 0 | 0 | 105 | 98 | 0 | 0 | 0 | 0 |
| Intergenic | 5,847,692 | 5,808,170 | 0 | 0 | 69,275 | 46,768 | 6 | 12 |
