# Supplementary material for: Metabolome and Transcriptome Reveal Novel Formation Mechanism of Early Mature Trait in Kiwifruit (Actinidia eriantha)
Source: Front Plant Sci. 2021 Nov 19;12:760496. doi: 10.3389/fpls.2021.760496 (PMC8640357; doi:10.3389/fpls.2021.760496)
Supplement: Supplementary file 10 [file Table_10.docx]

Supplementary Table 10 Twenty-four SNPs loci associated with early mature trait.

| Code | Gene ID | Variant sites | Reference genome | Second-generation sequencing test results | |
| --- | --- | --- | --- | --- | --- |
|  |  |  |  | GL2 | GL1 |
| 1 | DTZ79_00g02130 | 3952500 | C | T | T |
| 2 | DTZ79_02g02390 | 1965178 | C | T | C |
| 3 |  | 1965185 | T | G | T |
| 4 | DTZ79_02g11010 | 14374736 | G | A | G |
| 5 | DTZ79_03g06210 | 6321249 | T | C | T |
| 6 | DTZ79_03g09110 | 9310895 | C | T | T |
| 7 |  | 9312253 | G | A | A |
| 8 |  | 9312488 | A | G | A |
| 9 | DTZ79_04g01800 | 1880277 | G | T | T |
| 10 | DTZ79_06g06870 | 11793325 | C | T | C |
| 11 | DTZ79_07g07380 | 8217958 | A | G | G |
| 12 | DTZ79_08g09930 | 18499599 | C | T | C |
| 13 | DTZ79_08g18370 | 30278575 | T | C | C |
| 14 |  | 30278590 | A | G | G |
| 15 | DTZ79_10g07840 | 16471213 | C | T | C |
| 16 |  | 16474717 | A | T | A |
| 17 |  | 16475330 | C | A | A |
| 18 | DTZ79_13g06220 | 6053107 | G | T | T |
| 19 | DTZ79_19g16150 | 25720644 | C | T | T |
| 20 | DTZ79_22g06830 | 15246009 | T | A | A |
| 21 | DTZ79_22g07410 | 16006802 | T | G | G |
| 22 | DTZ79_23g10440 | 13849681 | C | T | T |
| 23 |  | 13849732 | T | A | A |
| 24 | DTZ79_27g04060 | 4096161 | G | C | G |
