# Supplementary material for: Metabolome and Transcriptome Reveal Novel Formation Mechanism of Early Mature Trait in Kiwifruit (Actinidia eriantha)
Source: Front Plant Sci. 2021 Nov 19;12:760496. doi: 10.3389/fpls.2021.760496 (PMC8640357; doi:10.3389/fpls.2021.760496)

Supplementary Figure 1 Dynamic change characteristics of ratio of endogenous hormone content of between 'Ganlv 2' and 'Ganlv 1'. (* represents that there are significant differences between different cultivars at same period, $P \leq 0.05$).

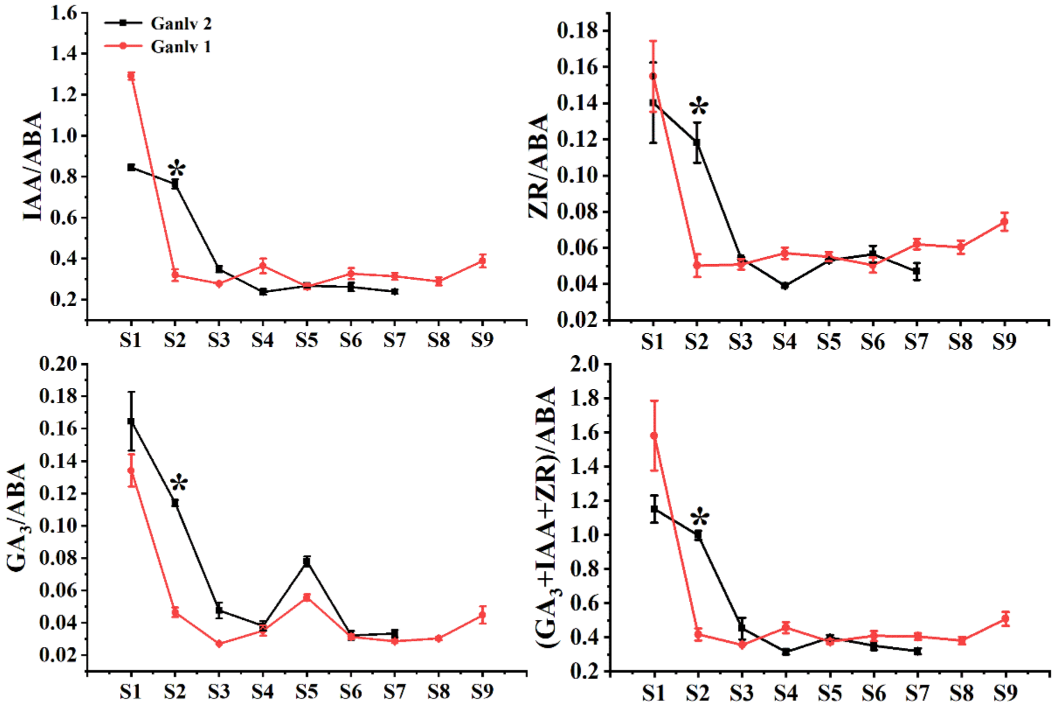

Supplement: Supplementary file 11 [file Image_1.pdf]
