# Supplementary material for: Metabolome and Transcriptome Reveal Novel Formation Mechanism of Early Mature Trait in Kiwifruit (Actinidia eriantha)
Source: Front Plant Sci. 2021 Nov 19;12:760496. doi: 10.3389/fpls.2021.760496 (PMC8640357; doi:10.3389/fpls.2021.760496)

Supplementary Figure 2 Partial least squares-discriminant analysis (OPLS-DA) of different comparison groups in POS (a) and NEG mode (b).

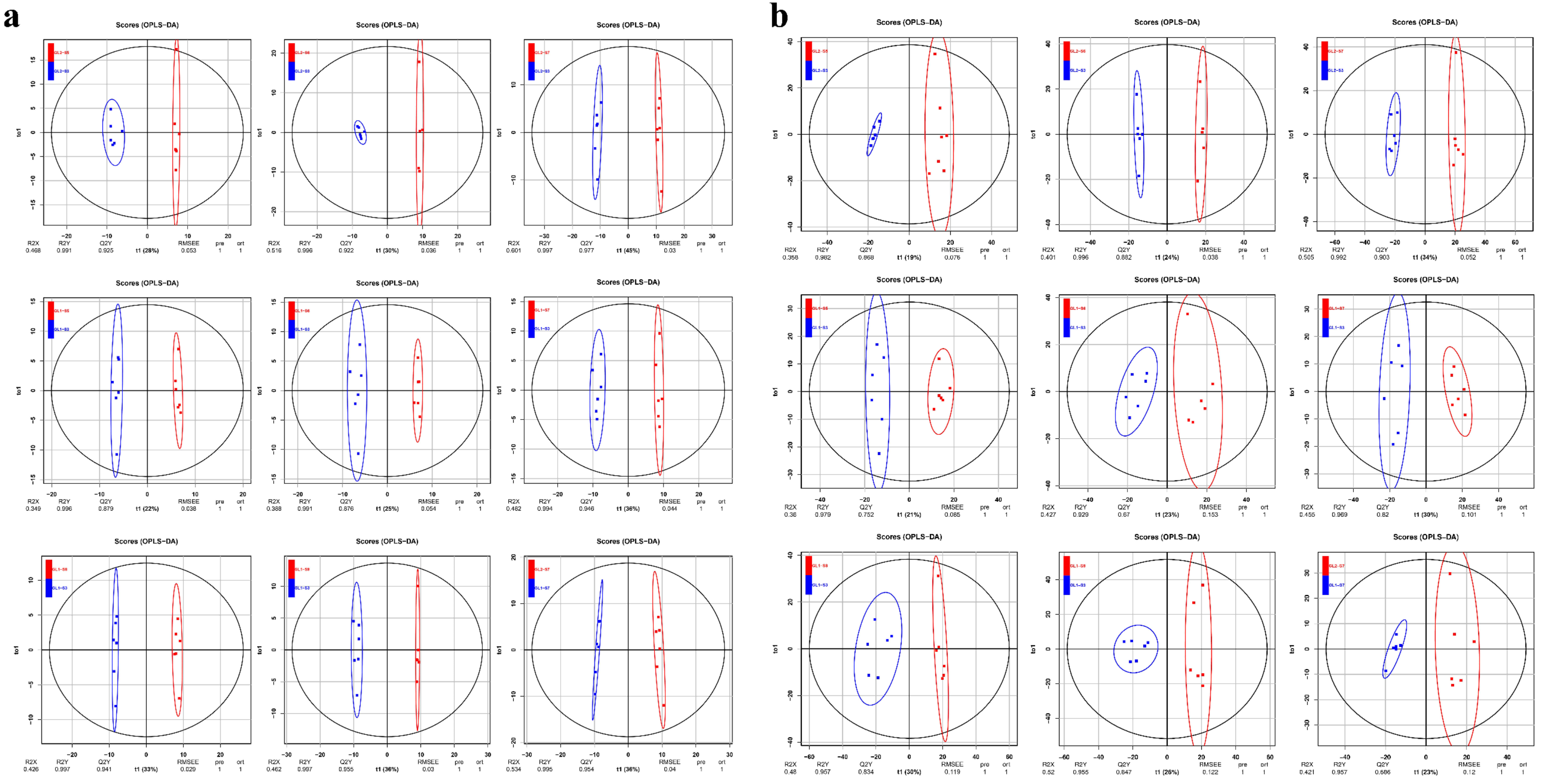

Supplement: Supplementary file 12 [file Image_2.pdf]
