# Supplementary material for: Metabolome and Transcriptome Reveal Novel Formation Mechanism of Early Mature Trait in Kiwifruit (Actinidia eriantha)
Source: Front Plant Sci. 2021 Nov 19;12:760496. doi: 10.3389/fpls.2021.760496 (PMC8640357; doi:10.3389/fpls.2021.760496)

Supplementary Figure 3 OPLS-DA model permutation test analysis of different comparison groups in POS (a) and NEG mode (b). The intersection point of the regression line on the ordinate is ≤ 0, which indicates that the prediction result of the model is reliable.

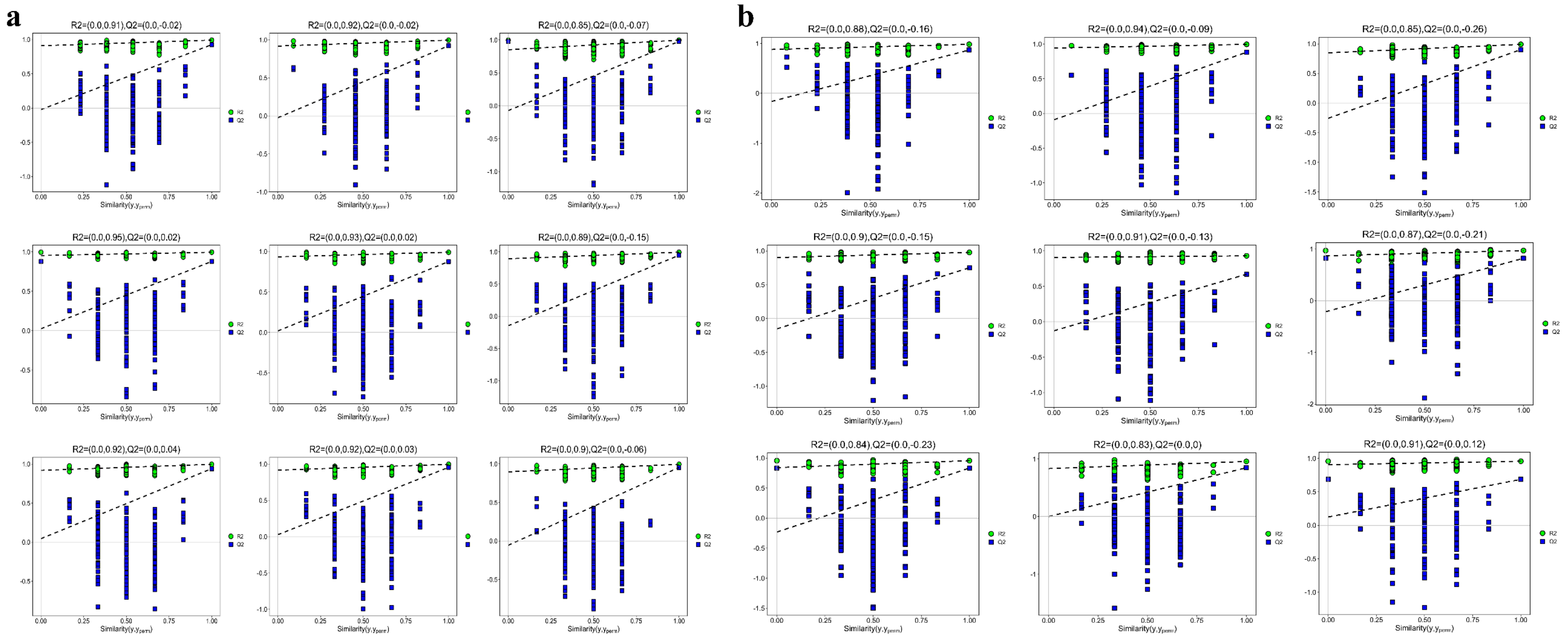

Supplement: Supplementary file 13 [file Image_3.pdf]
