# Supplementary material for: Metabolome and Transcriptome Reveal Novel Formation Mechanism of Early Mature Trait in Kiwifruit (Actinidia eriantha)
Source: Front Plant Sci. 2021 Nov 19;12:760496. doi: 10.3389/fpls.2021.760496 (PMC8640357; doi:10.3389/fpls.2021.760496)

Supplementary Figure 5 Analysis of DEGs related formation and regulation of early mature trait. Venn analysis of co-expressed DEGs (a), GO enrichment analysis (b), GO classification analysis (c), KEGG enrichment analysis (d), KEGG classification analysis (e).

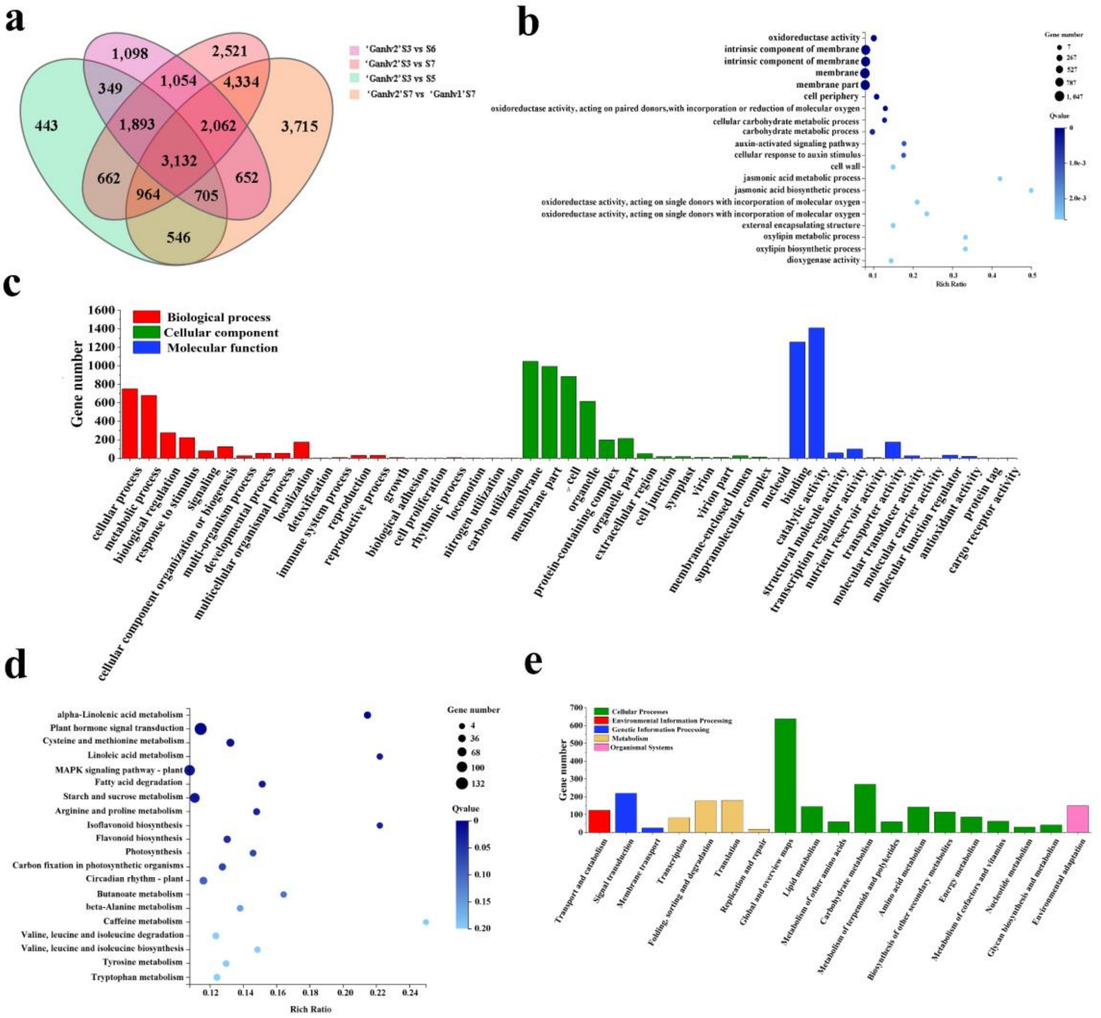

Supplement: Supplementary file 15 [file Image_5.pdf]
